# Immediate implant placement in infected sites using the “Roll BMP” technique: a 3-year case report with bioinformatic analysis

**DOI:** 10.3389/fdmed.2026.1750133

**Published:** 2026-03-30

**Authors:** Claudio Sotomayor Julio, Alfredo Torres

**Affiliations:** 1 Post-Graduate School, Faculty of Dentistry, University of Chile, Santiago, Chile; 2 AOS Training Institute, Santiago, Chile; 3 Department of Conservative Dentistry, Faculty of Dentistry, University of Chile, Santiago, Chile

**Keywords:** bioinformatics, BMP2, case report, immediate implant placement, infected site, soft tissue aesthetics

## Abstract

Immediate implant placement (IIP) in sites with infection and buccal bone wall deficiency remains a clinical challenge. This case report introduces the “ROLL BMP” technique, a flapless regenerative approach combining absorbable collagen sponge (ACS), biphasic calcium phosphate (BCP), and recombinant human Bone Morphogenetic Protein-2 (rhBMP2) to achieve simultaneous buccal bone reconstruction and soft-tissue preservation in type 2 post-extraction sockets. A healthy 56-year-old female presented with a root fracture and chronic infection at tooth 2.1. After atraumatic extraction and debridement, a 3.5 × 12 mm INNO sub implant (Cowellmedi, Busan, South Korea) was immediately placed. The biomaterial roll was created by layering BCP granules onto hydrated ACS, rolling it, and inserting it between the implant and soft tissue, followed by a 0.1 mL (1.5 mg/mL) rhBMP-2 injection (CowellBMP, Busan, South Korea). No flaps or membranes were required. Follow-ups at months (M) 3, 5, 12, 18, 24, and 36 demonstrated excellent peri-implant soft-tissue stability (Pink Esthetic Score 13 out of 14 at M36) and progressive trabecular bone maturation, accompanied by buccal cortical reconstruction on CBCT. In silico bioinformatic analysis identified BMP2, RUNX2, SPP1, and BGLAP as key osteogenic hub genes underlying bone repair and osseointegration, supporting the biological rationale for this approach. In conclusion, the “ROLL BMP” technique enables predictable, flapless IIP in compromised sockets while preserving both hard and soft tissues.

## 1 Introduction

Immediate implant placement (IIP) in infected extraction sites with buccal bone wall deficiency presents a significant clinical challenge. Success depends not only on achieving osseointegration but also on reconstructing soft and hard tissue contours that support optimal aesthetic outcomes (1, 2). These demands are particularly critical in the anterior maxilla, where biological and prosthetic factors converge under often unfavorable local conditions. Traditionally, such scenarios have been associated with higher implant failure rates and compromised esthetics due to bone and soft tissue loss (3–5).

Various techniques have been proposed to address these challenges. The “ice-cream cone” method, for instance, uses resorbable membranes to contain graft material in small to moderate dehiscences, but is limited in extensive or irregular defects where space maintenance is difficult (6). Guided bone regeneration (GBR) remains the gold standard, involving flap elevation, membrane placement, and particulate grafts to reconstruct the vestibular plate. While predictable, these procedures can disrupt the periosteal blood supply, compromise healing, and increase the risk of dehiscence or graft exposure (7, 8).

Additionally, GBR often requires multiple surgical stages, longer operative times, and delayed implant placement (7, 8) —factors that increase morbidity and reduce patient acceptance. In infected sockets or thin biotypes, complication rates and aesthetic unpredictability further rise (1, 2, 4, 9–11). These limitations have led to growing interest in minimally invasive, biologically driven alternatives that enhance regeneration while preserving vascular integrity. Advances in biomaterials and bioactive molecules have opened new pathways for predictable bone regeneration in complex cases. Recombinant human Bone Morphogenetic Protein-2 (rhBMP2) has emerged as a potent osteoinductive factor, capable of recruiting mesenchymal stem cells and promoting osteoblastic differentiation (12, 13). However, its clinical application in immediate post-extraction sites—particularly those with infection and buccal bone loss—remains limited and requires further validation.

Despite extensive evidence supporting BMP2's regenerative potential (12–18), no studies have integrated *in silico* bioinformatic analyses with clinical data in implant dentistry. Most studies focus on either molecular or clinical outcomes alone, leaving a gap in understanding how BMP2-mediated signaling translates into actual regenerative performance (12–16, 18). Predictive bioinformatics presents an opportunity to bridge this gap by identifying key genes, pathways, and biological processes that drive BMP2-induced bone formation and osseointegration. Integrating such analyses provides deeper mechanistic insight into clinical findings and a more biologically grounded evaluation of novel regenerative strategies (19).

**Figure 1:**
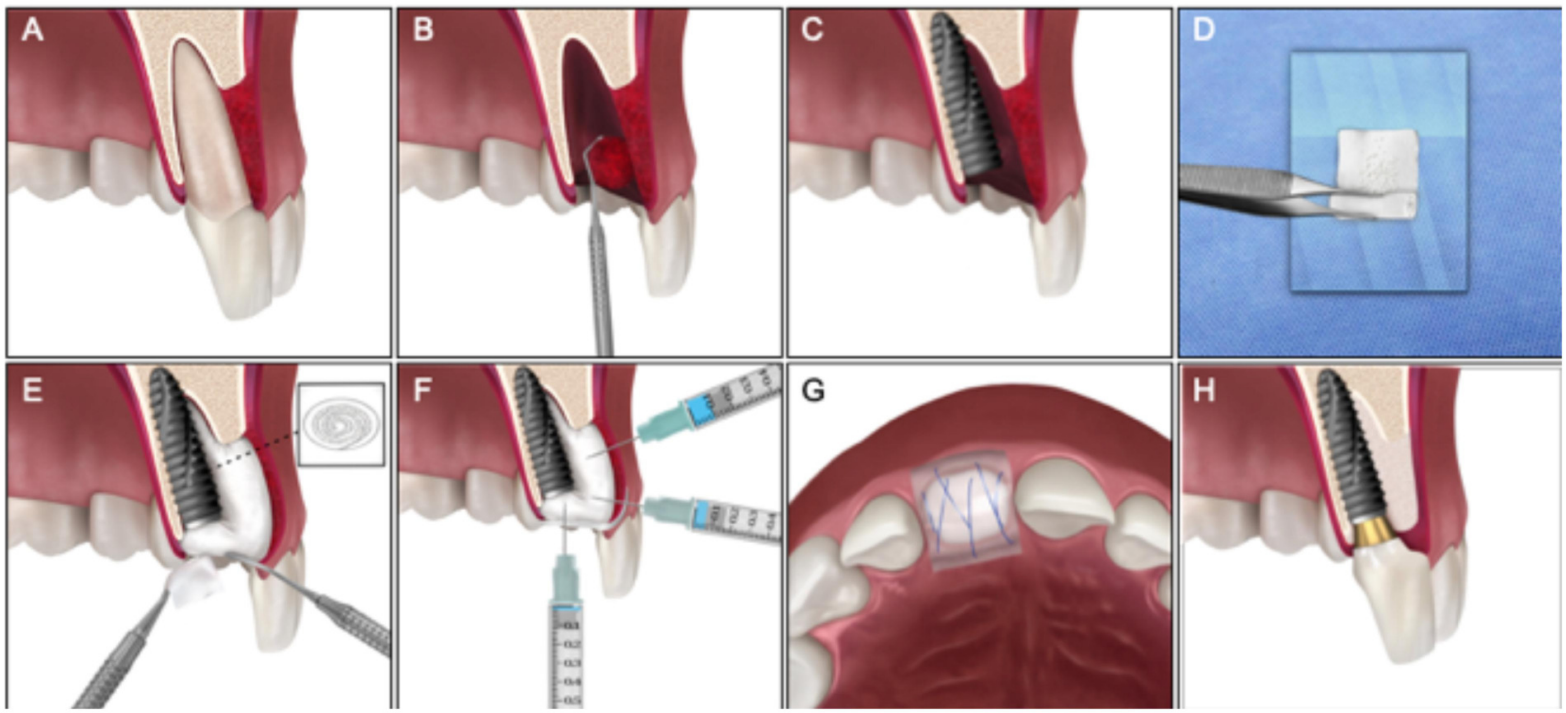
Schematic representation of the Roll-BMP technique for immediate implant placement in type 2 post-extraction sockets. **(A)** Compromised tooth with buccal plate deficiency and planned immediate implant placement. **(B)** Intra-alveolar debridement. **(C)** Implant placement within the prepared osteotomy, ensuring adequate primary stability and ideal three-dimensional positioning. Note the residual buccal gap between the implant surface and the inner aspect of the soft tissue. **(D)** Preparation of the absorbable collagen sponge (ACS) on a sterile field, over which biphasic calcium phosphate (BCP) granules are evenly distributed. The ACS is then rolled to form a cylindrical graft approximately 7–10 mm wide, enclosing BCP granules within its core, with the collagen surface oriented outward to face the soft tissues. **(E)** The Roll-BMP graft is packed evenly into the residual buccal gap to support the soft tissues. **(F)** Recombinant human bone morphogenetic protein-2 (rhBMP2) is reconstituted with 0.1 mL sterile saline and allowed to hydrate for 10–15 min. The hydrated BMP solution is subsequently injected into the Roll at three levels (I: coronal, II: middle, III: apical) to ensure uniform distribution throughout the grafted compartment. The final configuration shows the BMP-loaded ACS/BCP Roll surrounding the buccal aspect of the implant, providing structural support, space maintenance, and a dual-phase BMP release for enhanced tissue regeneration. **(G)** Occlusal view of the Roll on the buccal aspect of the implant and soft-tissue closure, which may be achieved by secondary intention or with tension-free 5/0 resorbable sutures if required. **(H)** Representation of the regenerated buccal bone plate with the Roll-BMP technique.

This case report introduces a flapless, immediate implant placement and regeneration technique—termed the “Roll BMP Technique”—for sites with severe vestibular bone loss and chronic infection. The approach combines an absorbable collagen sponge (ACS), biphasic calcium phosphate (BCP), and rhBMP2 to promote predictable regeneration of both hard and soft tissues without the need for membranes or flap elevation (Figure 1). Complemented by an *in silico* bioinformatic analysis of osteogenic pathways, this report integrates clinical innovation with molecular understanding, highlighting the therapeutic potential of BMP2-based regeneration in infected post-extraction sites.

## 2 Case report

**Figure 2:**
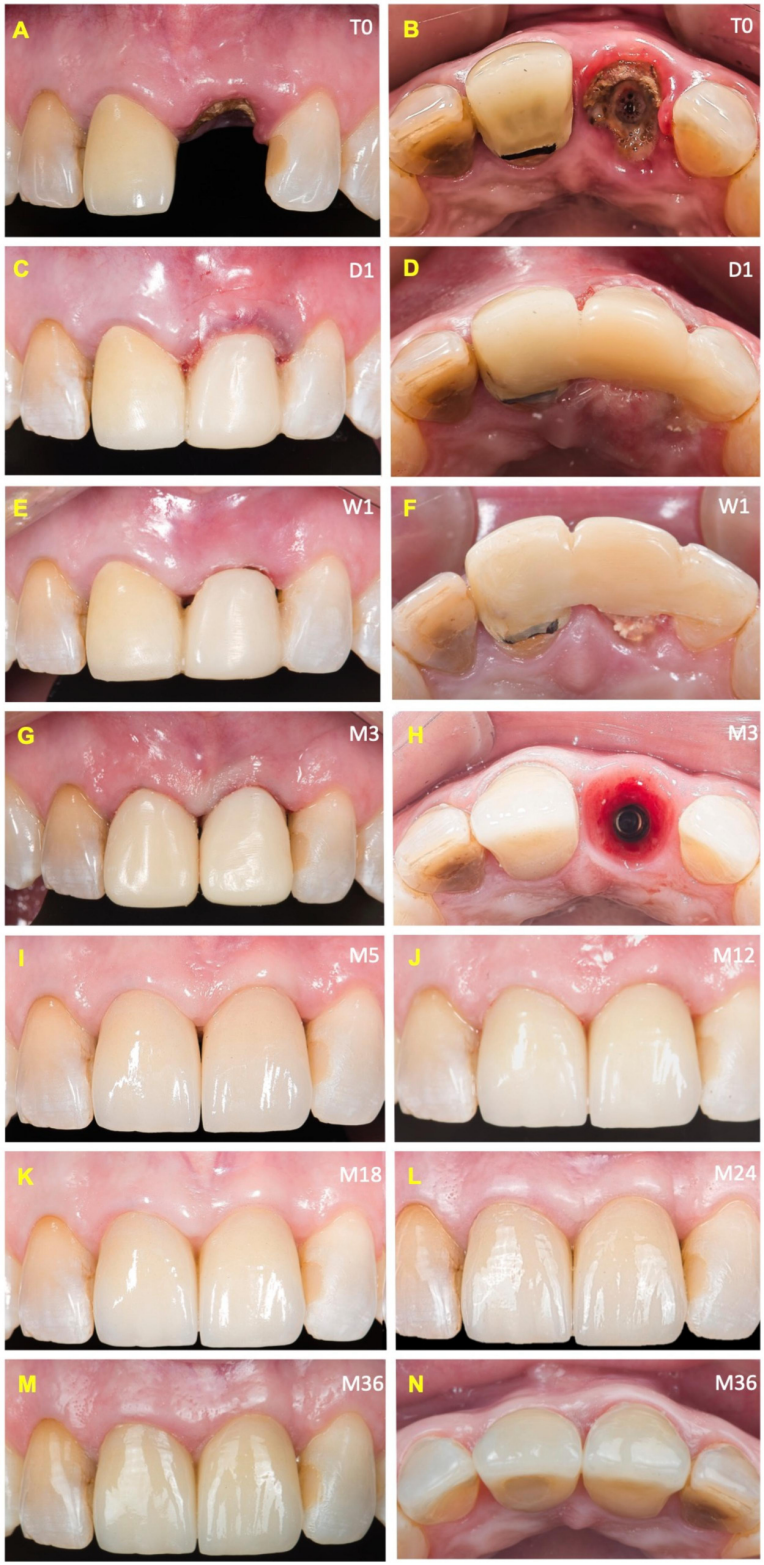
Clinical sequence of immediate implant placement and soft tissue evolution following the Roll-BMP technique. **(A,B)** Baseline clinical views (T0) showing gingival inflammation and coronal discoloration associated with a failing metal-ceramic restoration of tooth 2.1. The occlusal view reveals caries and a root fracture with loss of the buccal plate. **(C,D)** Immediate postoperative views (D1) following atraumatic extraction and immediate implant placement using the Roll-BMP protocol; the provisional restoration was bonded to adjacent teeth for stabilization. **(E,F)** One-week follow-up (W1) after application of the Roll-BMP technique; Clinical views showing early healing; the provisional restoration was bonded to adjacent teeth to ensure stabilization. **(G,H)** Three-month follow-up (M3) demonstrating soft tissue maturation and the emergence of keratinized mucosa; occlusal view shows healthy peri-implant mucosa surrounding the healing abutment. **(I)** Five-month follow-up (M5). Definitive custom zirconia abutment and lithium disilicate crowns fabricated at 2.1 and 1.1. **(J–M)** Clinical view at M12, M18, M24, and M36 after definitive prosthesis installation showing harmonious soft tissue integration, maturation, and improved papillary fill. **(N)** Occlusal view in long-term follow-up at 36 months (M36) demonstrating stable mucosal contours, sustained papilla architecture, and excellent aesthetic integration of the implant-supported restoration.

**Figure 3:**
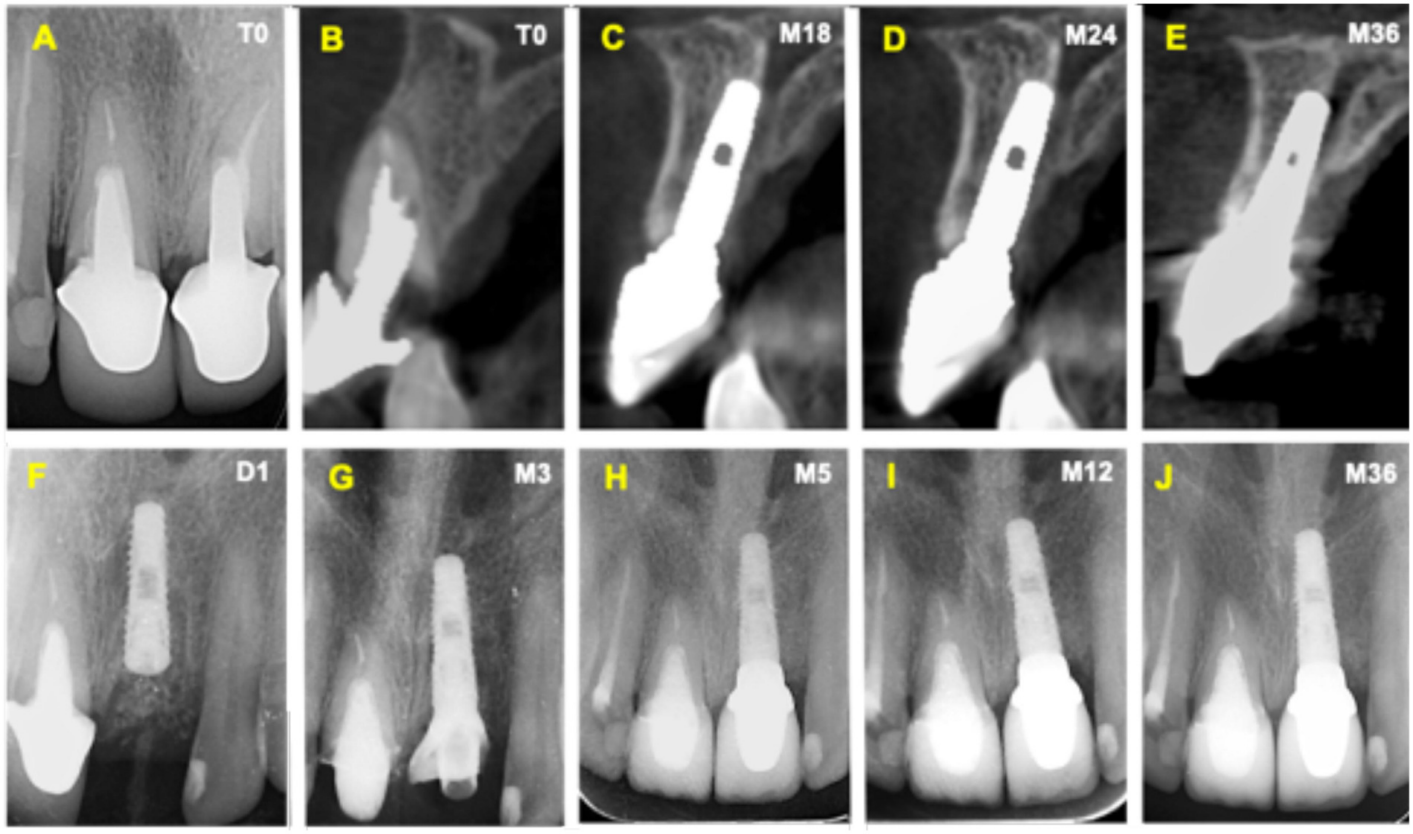
Radiographic and CBCT follow-up of immediate implant placement using the Roll-BMP technique. **(A)** Baseline periapical radiograph (T0) showing tooth 2.1 with advanced bone loss and root fracture. **(B)** Baseline sagittal CBCT section (T0) revealing a deficient buccal plate before extraction. **(C)** Sagittal CBCT view at 18 months (M18) demonstrating a newly formed buccal cortical plate with increased thickness (17.4 mm vertical measurement from implant apex to coronal level). **(D)** CBCT at 24 months (M24) showing maintained buccal bone contour and cortical density at the crestal level. **(E)** CBCT at 36 months (M36) confirming stable buccal bone regeneration and corticalization over time. **(F)** Immediate postoperative periapical radiograph (D1) displaying correct 3D implant positioning and presence of radiopaque BCP granules surrounding the implant. **(G)** Periapical radiograph at 3 months (M3) showing early bone formation with partial loss of visible BCP granules and initial trabecular organization. **(H)** At 5 months (M5), increased radiopacity and trabecular maturation are evident around the implant body. **(I)** At 12 months (M12), radiographic evidence of complete osseous integration and stable peri-implant bone density adapted to the prosthetic emergence profile. **(J)** At 36 months (M36), long-term stability with maintained bone levels and a consistent trabecular pattern confirming successful integration and remodeling of the regenerated site.

This case report has been written in accordance with the consensus-based clinical case reporting guideline development outlined in the CARE guidelines (20) (Supplementary Figure S1). A 56-year-old female presented with the chief complaint of increased mobility and discomfort in the upper left central incisor (tooth 2.1). No relevant medical or family history was reported. Clinical examination revealed a metal-ceramic single-unit prosthesis supported by a cast post and core in function for five years, showing increased mobility due to partial dislodgment. Gingival inflammation, secondary caries, and signs of vertical root fracture were evident (Figures 2A,B). A baseline (T0) periapical radiograph demonstrated a lack of continuity between the core and remaining dentin, as well as a suggestive radiolucency indicating a periapical lesion and root perforation. Cone-beam computed tomography (CBCT) imaging at baseline (T0) confirmed the absence of the buccal bone plate and the presence of a slight periapical lesion (Figures 3A,B). Notably, the axial inclination of the crown was inconsistent with the root axis, consistent with a vertical root fracture.

After a comprehensive diagnostic workup, including periapical radiographs and CBCT imaging, extraction and IIP were proposed using a novel surgical “ROLL BMP” technique, as described in Figure 1. The patient was fully informed regarding the innovative nature of the treatment, potential risks, and available alternatives. Written informed consent was obtained before treatment, including explicit consent for the use of clinical data and images for research and publication purposes. The procedure and follow-up were carried out in accordance with the principles of the Declaration of Helsinki and followed ethical clinical practice outside the context of a registered clinical trial.

Following the atraumatic extraction of tooth 2.1 and local debridement, an IIP protocol was carried out (D1) using a 3.5 × 12 mm Inno implant, positioned subcrestally with a 4-mm marginal gingival reference (Figures 2C, 3C), resulting in a subcrestal implant position (Figures 2C, 3C). The surgical site received a novel socket preservation protocol (Roll BMP technique) utilizing 0.1 g of BCP, 0.1 mg of rhBMP2 (Cowellmedi®), and an absorbable collagen sponge (ACS) matrix (Atelocare®) (Figures 1D–F). Briefly, the Roll BMP construct was prepared by evenly distributing beta-tricalcium phosphate (BCP) granules onto an absorbable collagen sponge (ACS) that had been hydrated with sterile saline. The composite was then gently rolled to form a cylindrical collagen–BCP construct, allowing intimate integration of the particulate graft within the collagen matrix before placement.

The site was covered with a resorbable pad (Oraid®) (Figures 1G, 2C,D), and a composite-retained provisional was bonded to adjacent teeth using Ribbond® reinforcement (Figures 2C,D, Supplementary Figure S2). Postoperative therapy medication regimen included amoxicillin 1 g every 12 h for 7 days, paracetamol 1 g every 12 h for 3 days, and ketoprofen 100 mg every 12 h for 3 days.

As seen in Figure 3, periapical imaging at day (D) 1 confirmed the correct 3D positioning of the implant and provided clear visualization of BCP granules within the socket. Radiographs taken at month (M) 3 revealed early radiolucent signs of bone maturation with loss of visible granules and initial osseous consolidation. By M5, the site exhibited increased radiopacity and advanced trabecular bone maturation. At M12, the radiographic appearance demonstrated stable bone density, evident trabecular patterns around the implant, and remodeling adapted to the prosthetic emergence profile.

CBCT scans at M18, M24, and M36 demonstrated consistent evidence of vestibular bone regeneration. At M18, a newly formed buccal cortical plate was noted, with increased density and thickness compared to the contralateral tooth. At M24-36, this corticalization was sustained and extended up to the crestal level, suggesting long-term stability of the augmented site (Figure 3).

The Pink Esthetic Score (PES) was assessed longitudinally to evaluate peri-implant soft tissue harmony (Supplementary Table S1). At M3, a score of 9 indicated early reestablishment of papillae and soft tissue architecture, albeit still maturing. At M5, following definitive prosthetic installation (custom zirconia abutment with lithium disilicate crown), the PES increased to 11 due to enhanced papillary fill and improved soft tissue color and texture. From M12 to M36, the PES remained stable at 13, with full integration of all soft-tissue parameters, except for a minor mesial papilla deficiency. Overall, the PES analysis confirmed a successful long-term aesthetic outcome (Figure 2) while acknowledging the multifactorial nature of PES outcomes, which are influenced by baseline tissue conditions, surgical and prosthetic factors, and operator-related variables.

The combination of clinical, radiographic, and PES outcomes supports the efficacy of the “ROLL BMP” technique in promoting stable hard and soft tissue regeneration in IIP protocols, even in sites compromised by infection and buccal bone deficiency.

## 3 Bioinformatics analysis

### 3.1 Identification of predictive coding-genes classification

Identifying and categorizing predictive coding genes represents a key step in elucidating complex biological processes and uncovering potential mechanisms underlying tissue regeneration and disease. To support the clinical rationale for the use of rhBMP2 in regenerative strategies for IIP, we conducted a comprehensive *in silico* analysis of the molecular landscape of bone regeneration and osseointegration.

The GÉNIE datamining tool (http://cbdm-01.zdv.uni-mainz.de/∼jfontain/g_search_adv_wp.php) (21) was utilized to identify genes with strong predictive associations to the terms “osseointegration” and “bone repair” (accessed March 30, 2025). Only studies based on human data were included, while orthologous and non-human datasets were excluded.

### 3.2 Protein–protein interaction (PPI) network construction, functional enrichment, and cluster analysis

To explore the biological context of the identified genes, functional enrichment analysis was conducted using ShinyGO v0.78 (http://bioinformatics.sdstate.edu/go/) (22). A protein–protein interaction (PPI) network was then generated using the STRING database (http://www.string-db.org; accessed March 30, 2025) (23) with a minimum confidence score of greater than 0.4, serving as the basis for subsequent clustering analyses. K-means clustering was applied to group genes with similar functional relationships, facilitating the identification of co-regulated modules, enriched pathways, and shared biological roles. These clusters were further analyzed to prioritize predictive coding genes with potential biomarker or therapeutic significance.

### 3.3 Identification of hub genes in the key modules

Hub genes were defined as those with the highest intramodular connectivity within each functional cluster. To identify them, topological properties of nodes in the PPI network were analyzed, including connectivity, path frequency, closeness, and neighborhood influence. Using Cytoscape (San Diego, CA, USA) (24) and the cytoHubba plugin, the top 10 hub genes were identified for each cluster. Seven nodal centrality algorithms—maximal clique centrality (MCC), maximum neighborhood component (MNC), degree, edge percolated component (EPC), closeness, radiality, and stress—were applied to enhance coverage and reduce redundancy (25). An UpSet diagram was generated to identify genes consistently ranked across algorithms, and the most robust candidates were defined as core hub genes. Functional characterization of these genes was performed using the UniProt database (26).

### 3.4 Statistical analysis

Data mining relevance was determined by the statistical prioritization of genes across the MEDLINE corpus. Coding genes showing significant enrichment were identified using a threshold of *p* < 0.01 at the abstract level and a false discovery rate (FDR) < 0.01 for gene selection. Fisher's exact test was used to evaluate the strength of association between individual genes and the target search terms.

Unsupervised k-means clustering was subsequently used to explore gene–gene interaction patterns by partitioning genes into clusters with similar multivariate interaction profiles, without assuming an underlying statistical distribution.

## 4 Results

### 4.1 Identification of predictive coding genes, protein-protein interaction (PPI) network construction, biological functions, and molecular functions analyses

**Figure 4:**
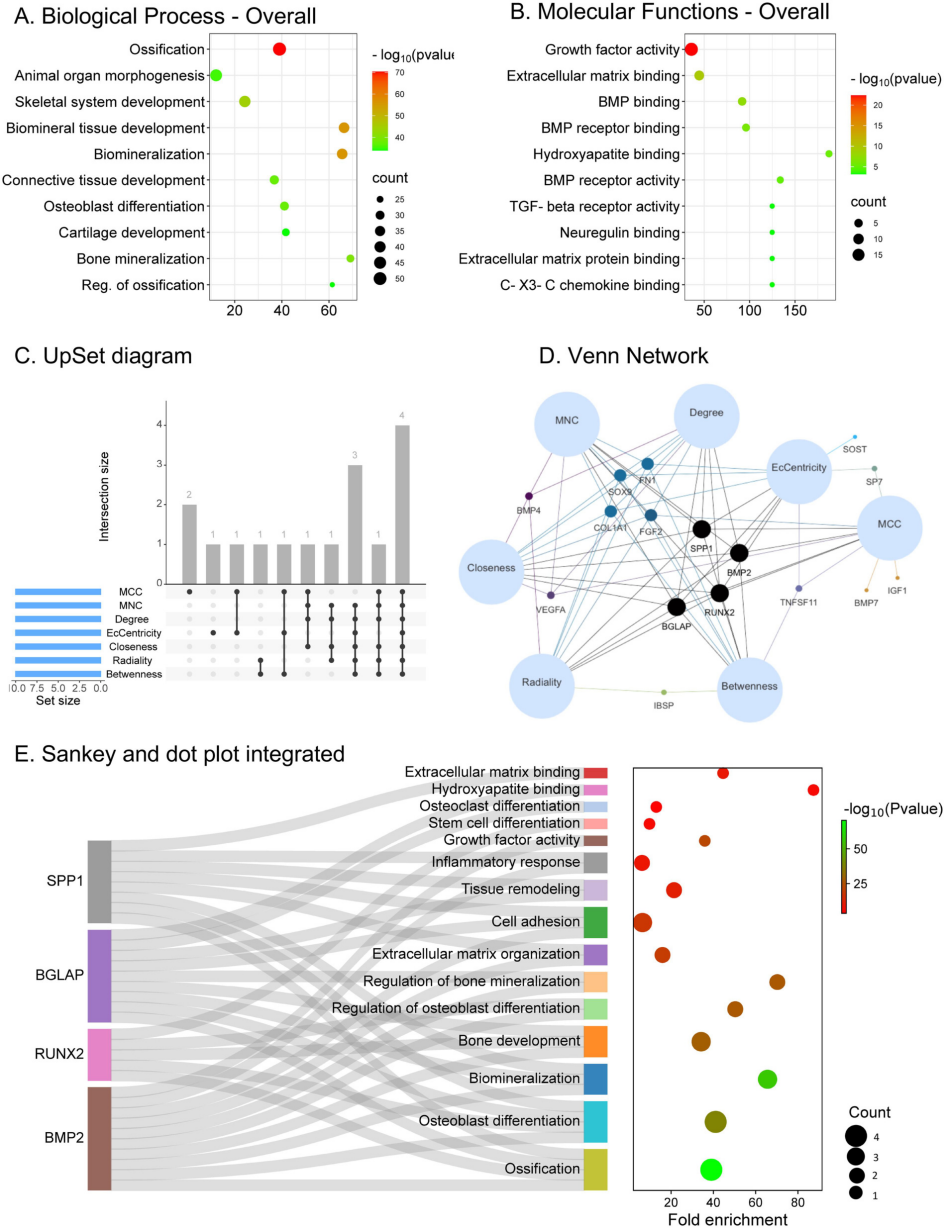
Bioinformatic enrichment analysis of predictive dataset associated with the query “bone repair” and “osseointegration” from the Génie datamining tool. **(A)** Functional annotation of enriched terms of gene ontology—biological process. **(B)** Functional annotation of enriched terms of gene ontology—molecular function. An adjusted *p* value < 0.05 was considered significant. **(C)** The UpSet diagram showed that the seven algorithms screened four overlapping hub genes. **(D)** Venn network showing four intersected hub genes: BMP2, RUNX2, BGLAP, and SPP1. **(E)** Sankey plot: Pathway Enrichment analysis of hub genes based on the Gene Ontology database.

Seventy-five protein-coding genes matching the PubMed query “osseointegration” and “bone repair were found as predictive dataset” (Supplementary Table S2). The PPI network comprising 75 protein-coding genes was generated using STRING (Supplementary Figure S2). Gene Ontology (GO) analyses were performed to assess Biological Processes (BP) and Molecular Functions (MF) associated with these genes (Figures 4A,B). GO-BP enrichment revealed predominant involvement in ossification, biomineralization, biomineral tissue development, osteoblast differentiation, and regulation of biomineralization (Figure 4A). In terms of GO-MF, the genes were mainly associated with growth factor activity, extracellular matrix binding, BMP binding, and Hydroxyapatite binding (Figure 4B).

### 4.2 Identification of hub genes

To further elucidate the functional mechanisms of the identified dataset, hub gene analysis was performed to identify key regulators involved in bone repair and osseointegration (Supplementary Figure S2). An UpSet diagram illustrated the overlap among hub gene sets across algorithms (Figure 4C). In contrast, a Venn diagram visualized the four intersecting hub genes: A consensus analysis across all methods revealed four central hub genes: Bone Morphogenetic Protein 2 (BMP2), Runt-related Transcription Factor 2 (RUNX2), Secreted Phosphoprotein 1 (SPP1, commonly known as Osteopontin), and Bone Gamma-Carboxyglutamate Protein (BGLAP, widely known as Osteocalcin). These genes are well-recognized for their pivotal roles in osteoinduction, matrix mineralization, and osteoblast differentiation (Figure 4D). The functional description and known biological roles of these *in silico*–identified hub genes related to bone repair and osseointegration are summarized in Supplementary Table S3 and depicted in Figure 4E.

### 4.3 Functional enrichment analysis of K-means–derived modules

K-means clustering of the PPI network revealed five significant functional modules; Gene Ontology–based enrichment analysis is further detailed in Supplementary Figure S3. Three distinct gene clusters were identified based on expression patterns and functional enrichment.

Cluster 1 was primarily associated with pro-inflammatory, angiogenic, and ECM remodeling activities. This cluster included IL1B, IL6, TGFB1, CXCL12, and PDGFB, reflecting a coordinated inflammatory and angiogenic response that initiates extracellular matrix remodeling and vascular invasion during early osseointegration. Immune and inflammatory responses, encompassing several pro-inflammatory cytokines (e.g., IL1B, IL6, TNF) and genes involved in neutrophil activation and leukocyte chemotaxis. Cluster 2 included genes related to Osteogenic differentiation and matrix organization. This cluster, containing BMP2, RUNX2, SPP1, and BGLAP, represented genes driving osteoblast differentiation, collagen maturation, and mineralized matrix deposition—hallmarks of the transition from provisional to lamellar bone, reflecting pathways linked to wound healing and osseointegration. Cluster 3 comprised genes involved in Phosphate and mineral regulation. This cluster comprised ALPL, BMP2, DMP1, and IBSP, highlighting their roles in phosphate metabolism, hydroxyapatite nucleation, and biomineralization processes essential for cortical bone consolidation. Together, these clusters delineate distinct yet interconnected biological processes underlying the inflammatory and reparative responses characteristic of peri-implant tissue remodeling (Supplementary Figure S3).

## 5 Discussion

IIP in Infected extraction sockets represent a biologically hostile microenvironment characterized by sustained inflammatory cytokine expression (e.g., IL-1β, IL-6, TNF-α), altered macrophage polarization, and impaired early angiogenesis, which not only compromise osteogenesis but may also translate clinically into accelerated bone resorption and loss of the buccal wall (2, 5, 9, 27). Conventional protocols recommend staged treatment with decontamination, grafting, and delayed implantation to minimize failure rates (2, 7). However, advances in biomaterials, biologically active molecules, and minimally invasive approaches have shifted this paradigm toward immediate rehabilitation, even in compromised sites (4, 5). The present case report introduces the “ROLL BMP” technique, a flapless regenerative approach for type 2 extraction sockets (27) that combines a collagen-based carrier, BCP, and rhBMP2, achieving stable bone and soft-tissue outcomes over three years.

The concept of IIP has evolved from strict selection criteria (3, 28) to broader inclusion supported by biomodulatory strategies (5). Studies show that, under controlled conditions and with adequate debridement (9, 11), implants in previously infected sockets can achieve survival rates comparable to those in pristine sites (1, 5, 29). Key factors are thorough granulation tissue removal, primary stability, and a biologically favorable healing environment (2). In this case, the implant was placed immediately after atraumatic extraction and meticulous debridement, achieving a subcrestal position to compensate for buccal loss and anticipated remodeling (1, 4, 5, 30). The flapless approach preserved the periosteal blood supply, minimized trauma, and improved soft-tissue stability, consistent with current minimally invasive trends that favor vascular preservation (29, 31).

Several techniques have been proposed for managing anterior defects with absent buccal bone plates, such as the “ice-cream cone” technique (6, 27) and other guided bone regeneration (GBR) approaches (32–35) designed for type 2 or 3 defects (27). While effective, these techniques often require flap elevation, membrane fixation, or the use of additional stabilization devices (32–35). The “ROLL BMP” technique challenges conventional GBR, which relies on flap elevation and membranes to separate healing compartments (7, 28, 36).

Although BCP granules and rhBMP-2 are widely used in regenerative procedures, their application has traditionally followed conventional GBR principles involving flap elevation and membrane-based compartmentalization (37, 38). In contrast, the “ROLL BMP” technique represents a biology-guided approach in which spatial control is achieved through graft architecture. The “ROLL BMP” technique addresses two primary challenges in immediate implantation: achieving predictable buccal reconstruction without a cortical plate and preserving mucogingival architecture without the use of flaps or membranes. It combines a resorbable ACS as a scaffold and carrier with BCP granules for volume stability and support, and a site-specific therapeutic dose of recombinant human BMP-2 (rhBMP-2), consistent with concentrations reported to be biologically effective in collagen-based carriers (14–16). As illustrated in Figure 1, the roll configuration positions collagen toward the soft tissue and BCP toward the defect core, promoting dual-phase regeneration—early soft-tissue stability and progressive bone formation —making it a minimally invasive alternative to conventional GBR.

The “ROLL BMP” technique addresses two primary challenges in immediate implantation: achieving predictable buccal reconstruction without a cortical plate and preserving mucogingival architecture without the use of flaps or membranes. It combines a resorbable ACS as a scaffold and carrier with BCP granules for volume stability and support, and a site-specific therapeutic dose of recombinant human BMP-2 (rhBMP-2), consistent with concentrations reported to be biologically effective in collagen-based carriers (14–16). As illustrated in Figure 1, the roll configuration positions collagen toward the soft tissue and BCP toward the defect core, promoting dual-phase regeneration—early soft-tissue stability and progressive bone formation —making it a valuable alternative.

The dual-phase BMP2 release—rapid from ACS and slower from BCP—ensures sustained local bioactivity during early healing, thereby fostering balanced remodeling and reducing resorption (14–16). Hydration and *in situ* injection of rhBMP2 enhance mesenchymal recruitment, differentiation, and angiogenesis (12), maximizing efficacy while minimizing losses and side effects. The flapless design minimizes morbidity and eliminates the need for membrane use, allowing for natural mucosal closure similar to biologically guided alveolar preservation (39).

BMP2 is among the most studied osteoinductive molecules, initiating the bone regeneration cascade (17, 18). It also modulates the immune microenvironment, promoting a Th2-dominant, tissue-reparative response while reducing pro-inflammatory activity (12). Thus, BMP2 functions not only as an osteogenic trigger but also as a local biological modulator that enhances regeneration (12). Its synergism with collagen matrices supports osteogenesis and angiogenesis by inducing osteoprogenitor differentiation and VEGF upregulation (40). In this case, rhBMP2 was used at a low dose (0.1 mg), following site-specific dosing principles to minimize edema or ectopic bone formation.

The bioinformatic analysis conducted in this study provides mechanistic and hypothesis-supporting insights into the observed clinical outcomes, reinforcing the biological plausibility of BMP2-mediated regeneration. BMP2, along with RUNX2, SPP1, and BGLAP, emerged as central hub genes within the protein–protein interaction (PPI) network, collectively orchestrating osteoblast differentiation, extracellular matrix mineralization, and bone remodeling (41–43). Enrichment analyses highlighted the activation of pathways associated with TGF-β signaling, Wnt regulation, and focal adhesion, which are essential for both hard and soft tissue integration. These findings underscore the molecular basis of the observed clinical results, linking the local regenerative process to key transcriptional regulators of bone formation, also known as osteogenesis. Nevertheless, these analyses should be interpreted as exploratory rather than predictive.

Soft tissue outcomes were assessed longitudinally using the Pink Esthetic Score (PES) as a within-case descriptive tool. In this clinical case, the “ROLL BMP” technique showed a progressive improvement and stability in PES from M12 through M36, highlighting favorable peri-implant soft tissue maturation while acknowledging the multifactorial determinants of PES outcomes, including surgical execution, implant positioning, biomaterial configuration, prosthetic management, and patient-specific healing capacity (5).

Regarding clinical significance, the long-term stability observed supports the hypothesis that controlled rhBMP2 delivery from a collagen–BCP scaffold can regenerate hard and soft tissues, even at infected sites. The ability to reconstruct the buccal plate and achieve esthetic outcomes without additional grafting highlights its potential. However, as a single case report, the results must be interpreted cautiously. Factors such as defect morphology, infection severity, and host response may influence outcomes (5, 29). Furthermore, regulatory and cost limitations may restrict clinical use. Controlled trials with histologic validation are required to confirm these findings and optimize dosage parameters.

A formal patient-reported outcome measure (PROM) was not collected, which represents a limitation of this case report. However, during follow-up, the patient reported satisfaction with functional and esthetic outcomes and absence of pain or procedure-related adverse events. Although these observations cannot replace validated PROMs, they provide supportive contextual information. Future studies should incorporate standardized patient-reported outcome instruments to assess patient-centered outcomes better.

The integration of bioinformatic analysis into this report adds a translational dimension, linking clinical outcomes to underlying molecular mechanisms. While the bioinformatic analysis provided a valuable mechanistic rationale for the clinical outcomes, as shown elsewhere (40), the predictive identification of osteogenic genes remains largely intuitive and hypothesis-generating rather than conclusive. Therefore, the proposed molecular framework should be interpreted cautiously and validated through further experimental and clinical studies with larger patient cohorts and standardized regenerative protocols.

Future perspectives drive the “ROLL BMP” technique as a potential shift toward a minimally invasive, biology-driven implant protocol capable of regenerating complex defects without extensive surgical manipulation. Future research should investigate quantitative histomorphometric outcomes, conduct long-term comparisons with GBR techniques, and perform transcriptomic analysis of peri-implant tissues exposed to BMP-based constructs. Refining biomaterial configuration—such as 3D-printed collagen or nanostructured carriers—could further optimize BMP kinetics and enhance predictability.

## 6 Conclusion

This three-year case report presents the clinical application of the Roll BMP technique, a biologically informed, minimally invasive regenerative approach for immediate implant placement in infected extraction sites with buccal bone wall loss. The flapless, membrane-free strategy enabled simultaneous management of hard and soft tissues while maintaining peri-implant tissue stability over time. Integration of bioinformatic analysis provided mechanistic, hypothesis-supporting insight into the biological processes underlying the observed clinical outcomes, reinforcing the regenerative rationale of the approach. Importantly, the clinical behavior observed should be interpreted within a multifactorial framework that includes surgical execution, implant positioning, biomaterial configuration, prosthetic management, and patient-specific healing capacity. Although limited by its nature as a single case report, these findings suggest that the “ROLL BMP” technique may represent a clinically applicable alternative to conventional staged regenerative approaches in selected compromised sites. Further controlled clinical studies and long-term evaluations are required to validate predictability and refine indications.

## Data Availability

The original contributions presented in the study are included in the article/Supplementary Material, further inquiries can be directed to the corresponding author/s.
