# Supplementary figures and images for: Immediate implant placement in infected sites using the “Roll BMP” technique: a 3-year case report with bioinformatic analysis
Source: Front Dent Med. 2026 Mar 30;7:1750133. doi: 10.3389/fdmed.2026.1750133 (PMC13070915; doi:10.3389/fdmed.2026.1750133)

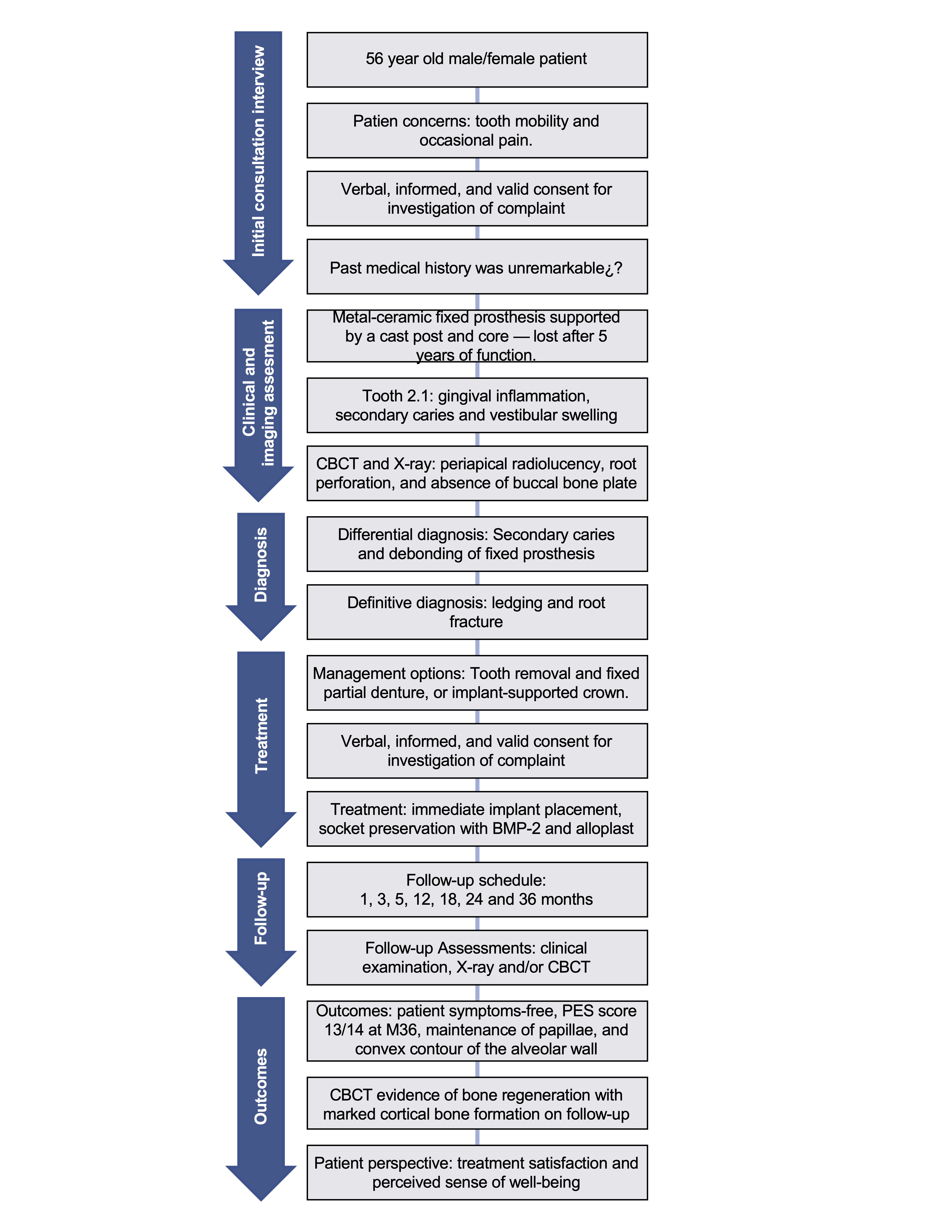

Supplement: Supplementary file 4 [file Image1.jpeg]

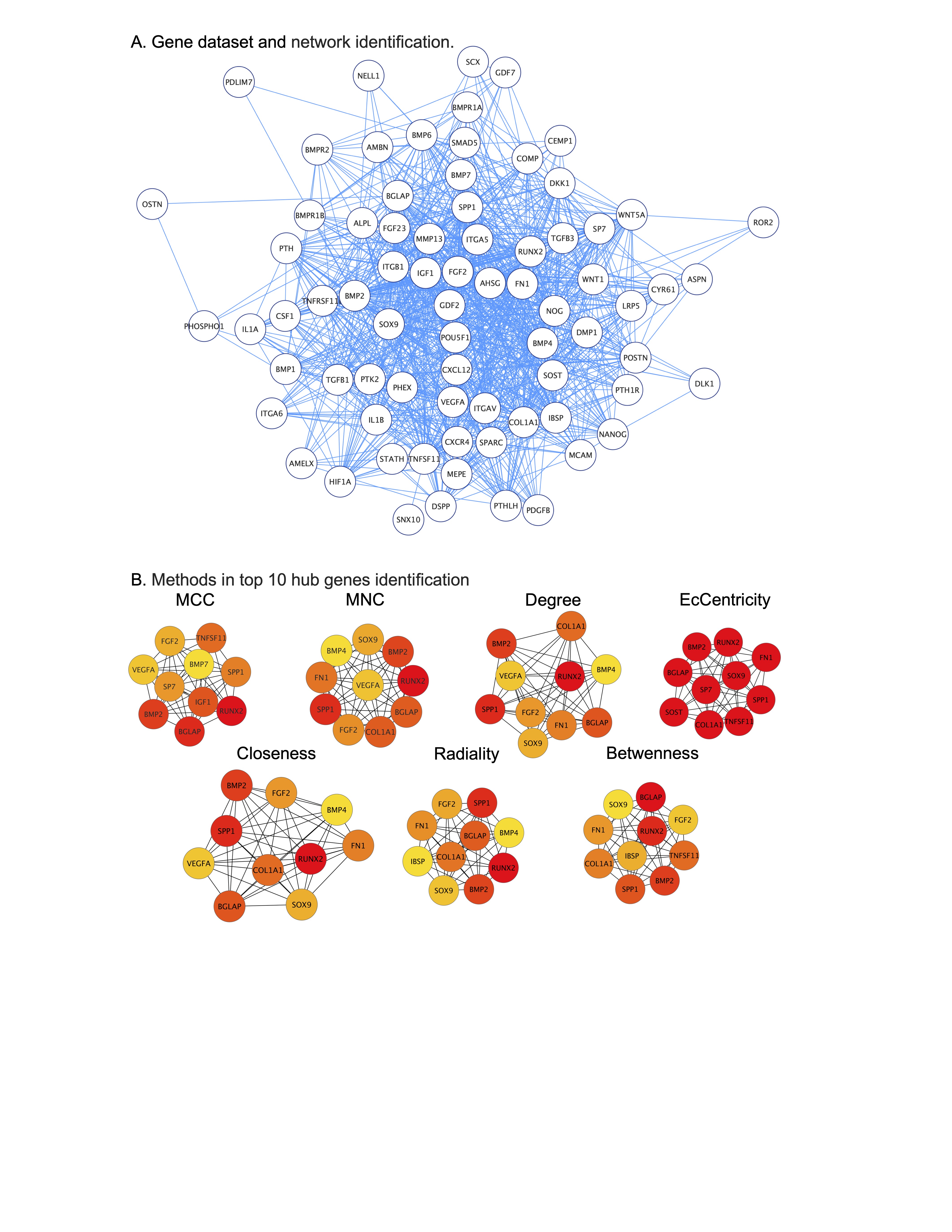

Supplement: Supplementary file 5 [file Image2.jpeg]

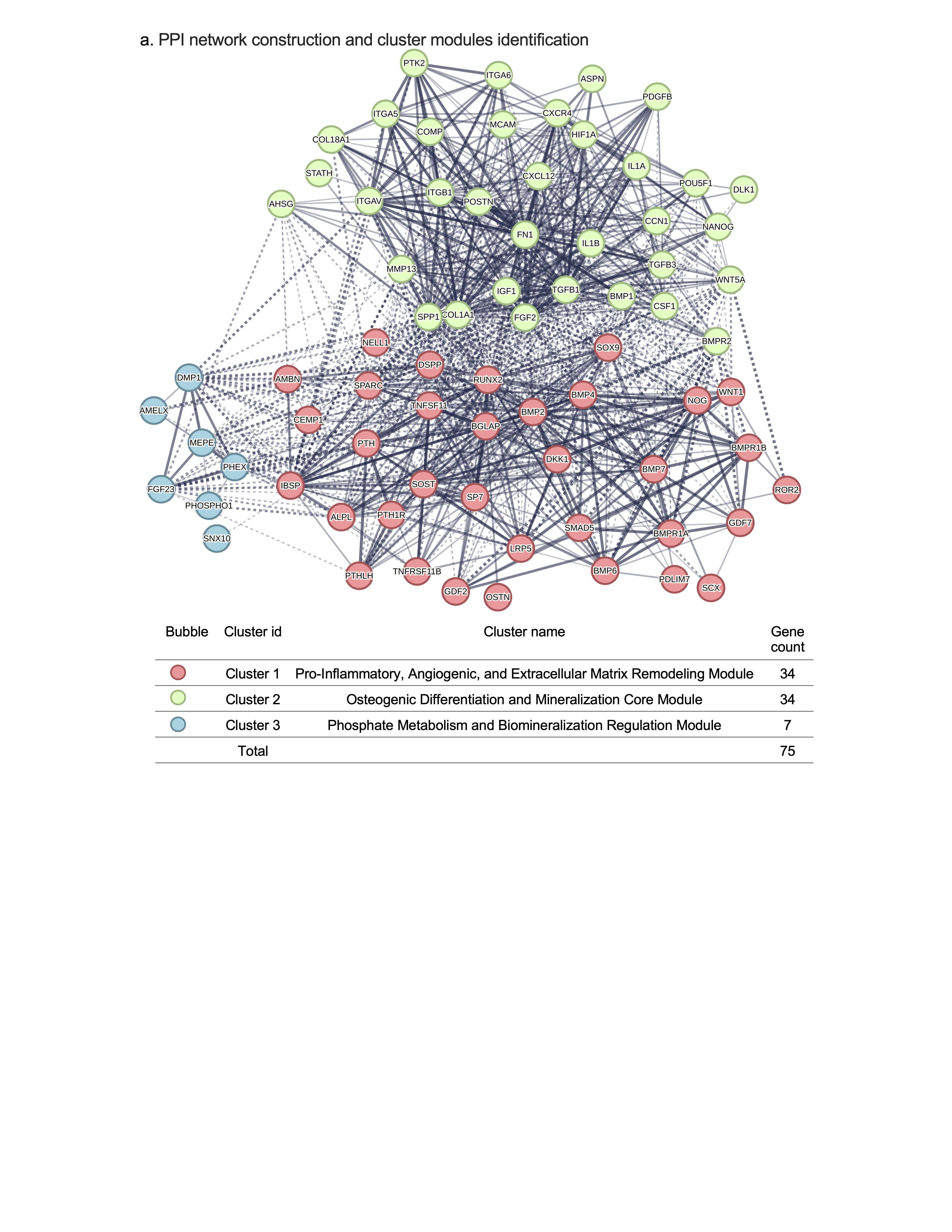

Supplement: Supplementary file 6 [file Image3.jpeg]
